# Real-world study and prognostic analysis of angioimmunoblastic T-cell lymphoma

**DOI:** 10.3389/fimmu.2024.1481301

**Published:** 2024-10-14

**Authors:** Suxiao Li, Xiaoyan Feng, Yunfei Song, Mengke Fan, Qingjiang Chen, Mingzhi Zhang, Xiaolong Wu, Meng Dong, Jieming Zhang, Lijuan Han, Xudong Zhang

**Affiliations:** ^1^ Department of Oncology, The First Affiliated Hospital of Zhengzhou University, Zhengzhou, Henan, China; ^2^ Department of Oncology, Lymphoma Diagnosis and Treatment Center of Henan Province, Zhengzhou, Henan, China; ^3^ Department of Oncology, Xinxiang Medical College, Xinxiang, Henan, China; ^4^ Henan Academy of Innovations in Medical Science, Zhengzhou, Henan, China

**Keywords:** angioimmunoblastic T-cell lymphoma, prognostic, treatment, prognostic model, outcomes

## Abstract

**Objective:**

To analyze the clinical prognostic factors and treatments for angioimmunoblastic T-cell lymphoma (AITL) and develop a novel prognostic model specifically for AITL.

**Method:**

We retrospectively analyzed 231 patients with AITL from the First Affiliated Hospital of Zhengzhou University. Patients were enrolled between January 2014 and July 2023. The primary end points were overall survival (OS) and progression-free survival (PFS).

**Result:**

The patients’ median age was 63 years, with 88.3% at an advanced stage (III/IV). The majority of patients (47.6%) received anthracycline-containing regimens, and there was no significant difference in survival compared with those treated with epigenetic-targeting and gemcitabine- containing regimens. The median PFS and OS were 6 and 17 months, respectively. In multivariate analysis, age >60 years, Eastern Cooperative Oncology Group performance status ≥2, elevated LDH, and splenomegaly were associated with inferior OS. Based on these four factors, a novel prognostic model (AITL model) was constructed that stratified patients into low‐, intermediate‐, and high‐risk groups, with 2-year OS estimates of 63.6%, 42.1%, and 18.6%, respectively.

**Conclusion:**

Currently, there is no consensus on the optimal initial therapy for AITL, and the efficacy of anthracycline-containing regimens remains suboptimal. The novel model developed herein demonstrates predictive significance for both OS and PFS, and exhibits better stratification and discrimination capabilities.

## 1 Introduction

Angioimmunoblastic T-cell lymphoma (AITL) is a distinct pathological subtype of peripheral T-cell lymphomas (PTCL), accounting for 15–20% of PTCL (1). AITL typically involves the expression of follicular helper T cell (TFH) markers, with positivity for two or more markers such as those encoded by *BCL6, CD10, CXCL13, PD-1*, or *ICOS*. Most patients are diagnosed at an advanced stage. AITL is characterized by systemic lymphadenomegaly, B symptoms, rash, serous cavity effusion, and splenomegaly. Currently, no standardized treatment exists for AITL; commonly used anthracycline-containing chemotherapy regimens have demonstrated poor long-term efficacy, with 5-year overall survival (OS) and progression-free survival (PFS) estimates of 32–41% and 18–38%, respectively (2). These statistics highlight the high mortality rate and rapid progression associated with AITL. Neoplasms remain the leading cause of death worldwide (3–8).

Few studies have attempted to identify clinicopathological and imaging-related adverse prognostic factors for AITL. Previous studies have found the presence of Epstein-Barr virus (EBV)-positive B cells in 66%–86% of AITL patients; these cells are detectable at an early stage post-diagnosis and potentially play a significant role in disease progression. Nevertheless, whether EBV status affects the prognosis of AITL is controversial (9–11). PET-CT is commonly used for tumor diagnosis and efficacy assessment, and studies have indicated that the tumor load at diagnosis has prognostic significance (12, 13). Spleen involvement has been shown to be an adverse prognostic factor in other lymphoma subtypes (14); however, whether splenomegaly is an independent poor prognostic factor for AITL remains uncertain.

Researchers have proposed several prognostic models for AITL, but their clinical utility remains controversial. For example, the Prognostic Index for PTCL-NOS (PIT) and its modified version (m-PIT) are primarily designed for PTCL-NOS, which exhibits distinct clinical and genetic characteristics from AITL. PIT incorporates age, performance status, lactate dehydrogenase (LDH), and bone marrow involvement, while the m-PIT replaces bone marrow invasion with a Ki-67 index of ≥80%. The Prognostic Index for AITL (PIAI) and AITL score were developed specifically for AITL. PIAI included (15), for the first time, extranodal involvement≥ 2, thrombocytopenia, and B symptoms, in addition to age >60 years and ECOG >2. However, PIAI only categorizes patients into two risk strata. The AITL score, proposed in the T-cell Project (TCP), has been validated in a limited dataset of 96 patients (16). Given the limitations of existing models, there is an urgent need for AITL-specific prognostic models capable of accurately identifying high-risk patients and assisting clinical decision-making. Therefore, the objective of this study was to further characterize the clinical features of AITL and identify critical prognostic factors at the time of diagnosis.

## 2 Materials and methods

### 2.1 Patients

For this study, 231 patients diagnosed with AITL between 2014-01-01 and 2023-07-01 at the First Affiliated Hospital of Zhengzhou University were enrolled. Clinical data were retrospectively analyzed, including clinical symptoms, laboratory findings, pathology, bone marrow involvement, treatments, and survival outcomes. The cohort comprised 155 males and 76 females, with a median age of 63 years (range, 26–94 years). Of these, 204 patients (88.3%) were classified as stage III/IV. Inclusion criteria were as follows: (1) pathologically confirmed diagnosis of AITL according to the World Health Organization (WHO) classification and (2) available clinical data, including baseline information for staging, treatment regimens, efficacy evaluation, and follow-up. Exclusion criteria were as follows: (1) acute myocardial infarction or cerebral infarction within 6 months; (2) uncontrolled hypertension and symptomatic arrhythmia; and (3) pregnancy or lactation. This study was approved by the Medical Ethics Committee of the First Affiliated Hospital of Zhengzhou University, Henan Province (2022-KY-0869-001) and patients provided informed consent.

In our cohort, patients were primarily treated with CHOP-like chemotherapy regimens, while other common regimens included those containing gemcitabine or epigenetic drugs. These chemotherapeutic regimens were administered according to standard protocols regarding dose, timing, duration, and cycle (17–19).

### 2.2 Statistical analysis

A retrospective analysis of the cohort (n=231) was conducted to characterize the clinicopathologic features and identify prognostic factors for proposing a new prognostic model. The primary end points of the study were OS and PFS, which were calculated using the log-rank test and Kaplan–Meier survival curves. ROC analysis was used to determine the ideal critical values for PET/CT metabolic parameters (SUVmax). To identify the prognostic factors, univariate and multivariate analyses were performed using Cox proportional hazards regression models. Significant covariates (with p < 0.05) were incorporated into the multivariate analyses.

## 3 Results

### 3.1 Clinicopathological features of patients with AITL

**Table 1** summarizes the clinical characteristics of 231 patients with AITL. The median age was 63 years (range 26–49). Most patients were male (67.1%). A total of 88.3% (204/231) of patients presented with advanced-stage disease (III/IV), while 58.9% (136/231) and 9.5% (22/231) had splenomegaly and hepatomegaly, respectively. Additionally, extranodal involvement at two or more sites was observed in 19.9% (46/231) of patients, and bone marrow involvement was noted in 27.9% (64/229). Furthermore, rash occurred in 16.9% (39/231), joint pain in 5.2% (12/231), and serous cavity effusion in 51.9% (120/231) of patients. Peripheral blood EBV-DNA ≥500 copies/mL was detected in 42.2% (54/128) patients. EBER- status were detected by ISH in 203 patients, with 65.5% (103/203) expressing EBV-infected B cells (EBER+).

**Table 1:** Clinicopothological features of 231 patients with AITL.

		OS		OS	
		Univariate analysis		Multivariate analysis	
Variables	N(%)	P	HR(95%CI)	P	HR(95%CI)
Age, median (range)	63(26–94)				
Age>60 years	131/231(56.7%)	0.005	1.599(1.151,2.221)	0.024	2.321(1.117,4.825)
Male	155/231(67.1%)	0.137	1.305(0.919,1.854)		
Stage III~IV	204/231(88.3%)	0.127	1.537(0.885,2.670)		
ECOG≥2	79/231(34.2%)	0.000	1.931(1.384,2.694)	0.044	2.443(1.026,5.815)
B symptoms	150/231(64.9%)	0.203	1.248(0.887,1.756)		
WBC<10000/mm^3^	42/231(18.2%)	0.496	1.147(0.772,1.705)		
Platelets<100000/mm^3^	43/231(18.6%)	0.148	1.357(0.897,2.053)		
Anemia	142/231(61.5%)	0.021	1.459(1.058,2.010)	0.559	0.800(0.378,1.691)
NLR<4.635	119/231(51.5%)	0.029	1.429(1.038,1.967)	0.187	0.597(0.278,1.283)
LDH>ULN	144/221(65.2%)	0.001	1.880(1.295,2.728)	0.049	2.560(1.005,6.516)
Alb<3.5 g/dL	108/230(47.0%)	0.001	1.747(1.263,2.416)	0.261	1.524(0.732,3.173)
Hypergammaglobulinemia	75/228(32.9%)	0.310	1.192(0.849,1.675)		
β2M>ULN	131/207(63.3%)	0.000	1.997(1.372,2.906)	0.317	0.590(0.209,1.660)
Eosinophil<0.02	193/231(83.5%)	0.000	0.487(0.328,0.723)	0.461	1.561(0.478,5.102)
Extranodal sites≥2	46/231(19.9%)	0.460	1.141(0.804,1.619)		
Bone marrow involvement	64/229(27.9%)	0.154	1.304(0.905,1.877)		
Pneumonia	199/231(86.1%)	0.532	1.166(0.720,1.888)		
Splenomegaly	135/231(58.4%)	0.022	1.470(1.057,2.043)	0.013	3.117(1.267,7.664)
Hepatomegaly	22/230(9.6%)	0.331	1.285(0.775,2.132)		
Rash	39/231(16.9%)	0.382	1.199(0.798,1.801)		
Joint pain	12/230(5.2%)	0.434	1.309(0.667,2.571)		
Serous cavity effusion	120/231(52.4%)	0.024	1.453(1.051,2.008)	0.284	1.495(0.717,3.117)
Ki67>45%	55/230(23.9%)	0.005	1.726(1.181,2.522)	0.792	0.894(0.388,2.058)
SUVmax>12	81/138(58.7%)	0.036	1.628(1.032,2.569)	0.183	1.716(0.775,3.801)
CD20+	124/225(55.1%)	0.026	0.686(0.493,0.955)	0.087	0.557(0.285,1.089)
CD30+	102/111(91.9%)	0.987	0.995(0.536,1.845)		
DBIL>6.8	52/231(22.5%)	0.002	1.782(1.236,2.569)	0.346	0.637(0.249,1.628)
EBER-ISH	133/203(65.5%)	0.983	1.004(0.699,1.442)		
EBV-DNA ≥500 copies/mL	54/128(42.2%)	0.011	1.763(1.141,2.726)	0.803	0.899(0.390,2.074)

NLR, neutrophil to lymphocyte ratio; SUVmax: standard uptake value of lymph node in PET-CT. ULN: Upper limit of normal. LDH >ULN: the elevated LDH, LDH >245 IU/L. Splenomegaly: the long diameter of the spleen >12 cm on US or CT, the vertical diameter of spleen >13 cm on PET/CT. β2M: β2 microglobulin.

### 3.2 Treatment regimens and outcome

Among the cohort, 100 patients (43.2%) received anthracycline-containing chemotherapy (CHOP-like), including CHOP (n = 53), CHOPE (n = 11), and other anthracycline-based regimens. Forty-three patients (18.6%) received GDP-like regimens (e.g., GDP, GDPT), while 40 patients (30.7%) were treated with epigenetic-modifying therapies (PET-like), including PET (n=9) and CPET (n=31) regimens. Compared with CHOP-like regimens, PET-like and GDP-like did not improve the outcomes (all P > 0.05, **Table 2**). To assess the efficacy of anthracyclines, epigenetic agents, and gemcitabine, we categorized all patients into an anthracyclines (n=100) and non-anthracyclines (n=110) group; epigenetic (n=71) and non-epigenetic (n=139) group; and gemcitabine (n=51) and non-gemcitabine (n=159) group. Additionally, 21 patients (9.1%) received only symptomatic treatment post-diagnosis. Only 9 patients underwent consolidative autologous stem cell transplantation (ASCT).

**Table 2:** Common chemotherapeutic regimens in patients with AITL in the cohort.

Characteristics		N	P value (OS)	P value (PFS)
Category 1	CHOP-like	100	0.589	0.435
	GDP-like	43		
Category 2	CHOP-like	100	0.482	0.754
	PET-like	40		
Category 3	GDP-like	43	0.818	0.708
	PET-like	40		
Category 4	CHOP	53	0.545	0.943
	CHOPE	11		
Category 5	Epigenetics	71	0.743	0.705
	Non-epigenetics	139		
Category 6	Anthracycline	100	0.681	0.891
	Non-anthracycline	110		
Category 7	Gemcitabine	51	0.985	0.941
	Non-gemcitabine	159		

CHOP, Cyclophosphamide + Hydroxydoxorubicin + Oncovin + Prednisone; CHOPE, CHOP + Etoposide; GDP, Gemcitabine + Cisplatin+ Dexamethasone; GDPT, GDP + Thalidomide; PET, Prednisone + Etoposide + Thalidomide; CPET, Cedaramine + Prednisone + Etoposide + Thalidomide.

**Figure 1:**
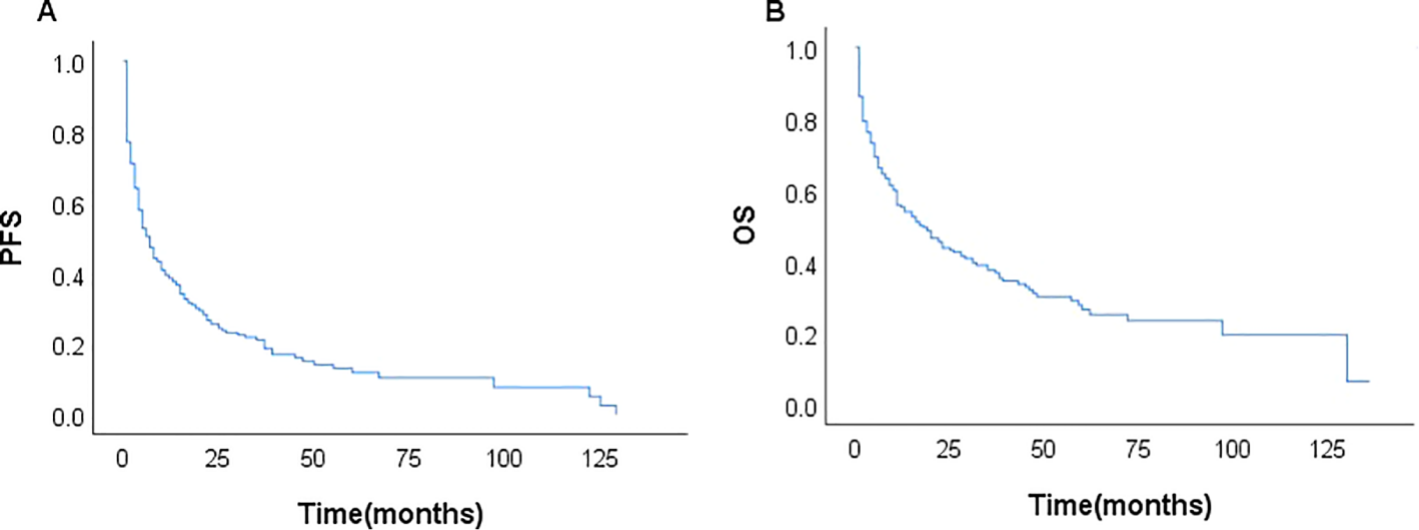
Kaplan–Meier curves of the entire study cohort (n=231); PFS **(A)** and OS **(B)**.

**Figure 2:**
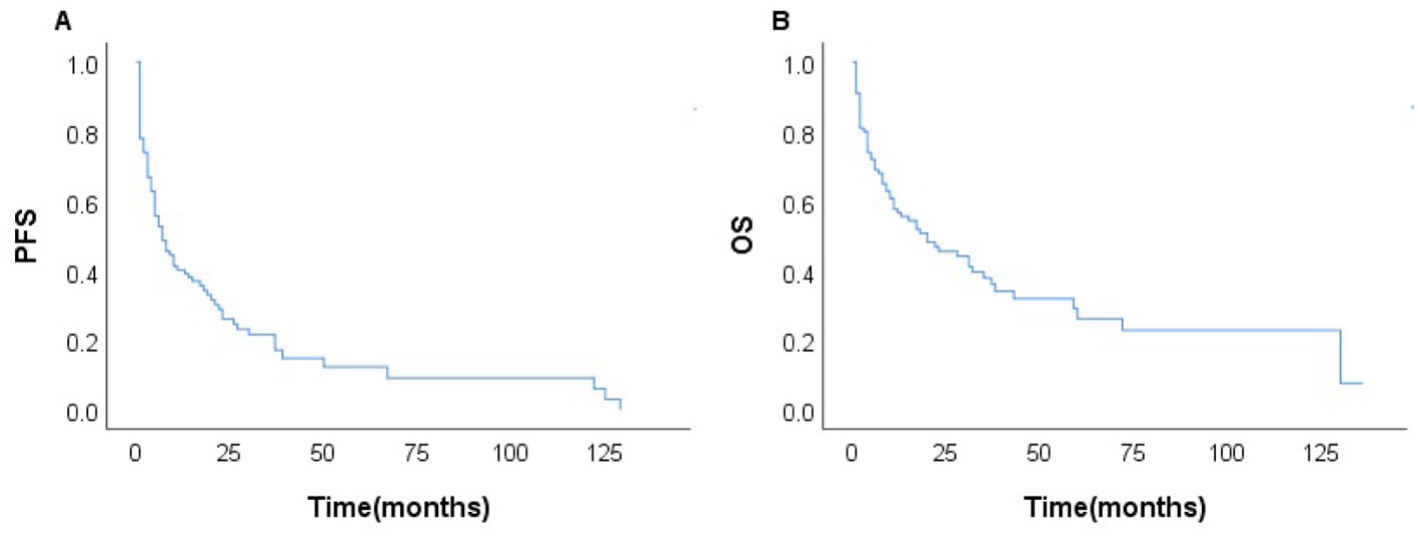
Survival of the patients with AITL treated with CHOP-like regimens (n=100); PFS **(A)** and OS **(B)**.

**Figure 3:**
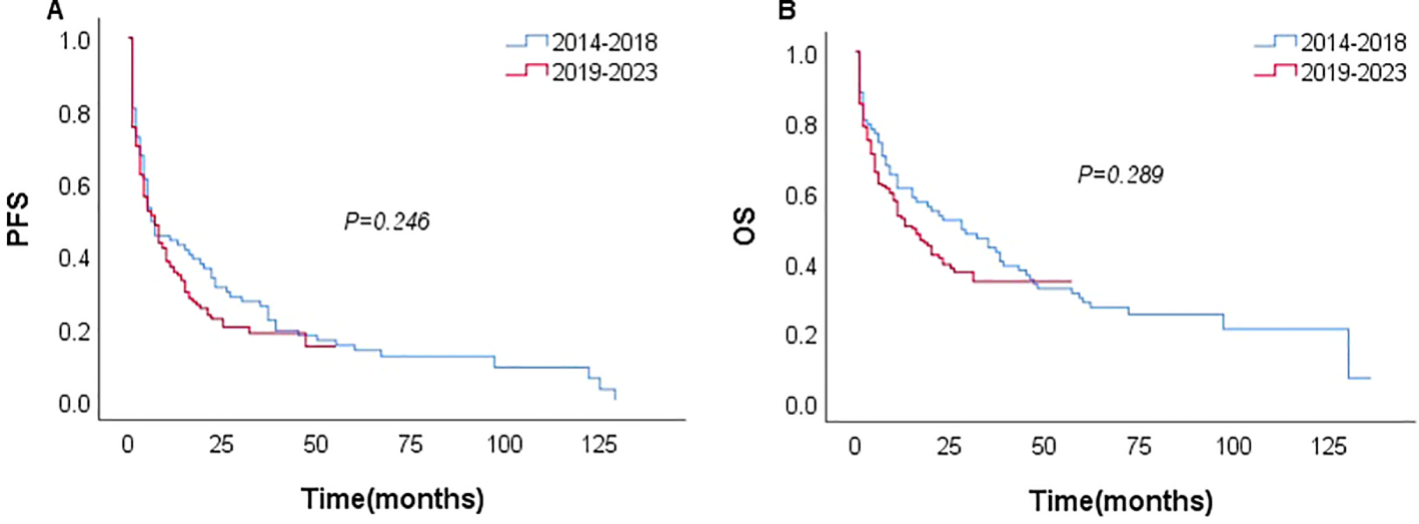
Survival at different periods (n=231); PFS **(A)** and OS **(B)**.

Among the 231 patients in this study, there were 121 deaths recorded, with 102 of these deaths occurring within the first year after diagnosis. The median follow-up duration was 42 months (range, 1–135 months). The 3- and 5-year OS rates for the entire group were 37.7%, and 26.6%. The 3–, and 5–year PFS rates were 21.1% and 11.9%, respectively. The median PFS and OS were 6 and 17 months, respectively (**Figure 1**). In patients treated with CHOP-like therapy, the median PFS and OS were 7 and 20 months, respectively, with 5-year PFS and OS rates of 12.3% and 26.2% (**Figure 2**). For patients who received anthracycline-containing chemotherapy regimens with or without etoposide, there was no significant difference in OS and PFS (**Table 2**). Notably, there was no significant difference in OS or PFS among patients treated with anthracycline-, gemcitabine- or epigenetic agent- containing regimens (all P>0.05). Additionally, there was no significant difference in OS or PFS between patients treated during the periods 2014-2018 (n=77) and 2019-2023 (n=154; **Figure 3**).

### 3.3 Prognostic index

The prognostic value of the PIT, m-PIT, PIAI, and AITL score were assessed in this study. Patients were categorized into 4 risk groups based on PIT, 3 risk groups based on AITL score and m-PIT, and 2 risk groups based on PIAI (**Table 3**). Patients classified as high-risk based on PIT (27.5%, 60/218), m-PIT (21.2%, 47/222), PIAI (66.2%, 153/231), and AITL scores (42.6%, 75/176) had 4-year PFS estimates of 6.6%, 6.4%,11.5% and 3.9%, respectively, and 4-year OS estimates of 22.3%, 23.8%, 26.2% and 22.3%, respectively (all P < 0.05).

**Table 3:** Comparison of established prognostic indices.

Prognostic index	N(%)	2-y OS	4-y OS	2-y PFS	4-y PFS
PIT					
Low-risk (0)	24/218 (11.0)	73.7	56.1	45.1	32.9
low-int risk (1)	64/218 (29.4)	60.5	37.2	43.8	28.2
High-int risk (2–3)	70/218 (32.1)	39.6	20.6	20.1	3.3
High-risk (3–4)	60/218 (27.5)	22.3	22.3	6.6	6.6
m-PIT					
Low-risk (0–1)	110/222 (49.5)	57.7	38.5	36.5	23.5
Int-risk (2)	65/222 (29.3)	35.0	21.6	16.8	5.6
High-risk (3–4)	47/222 (21.2)	27.8	23.8	12.8	6.4
PIAI					
Low-risk (0–1)	78/231 (33.8)	50.0	37.3	32.7	21.9
High-risk (2–5)	153/231 (66.2)	40.7	26.2	21.8	11.5
AITL score					
Low-risk (0–1)	42/176 (23.9)	65.9	37.1	39.0	24.8
Int-risk (2)	59/176 (33.5)	45.1	30.1	21.6	13.7
High-risk (3–4)	75/176 (42.6)	34.1	22.3	20.3	3.9
AITL model					
Low-risk (0–1)	68/221 (30.8)	63.6	49.8	42.4	29.8
Int-risk (2–3)	123/221 (55.7)	42.1	25	23.4	10.7
High-risk (4)	30/221 (13.6)	18.6	18.6	0	0

**Figure 4:**
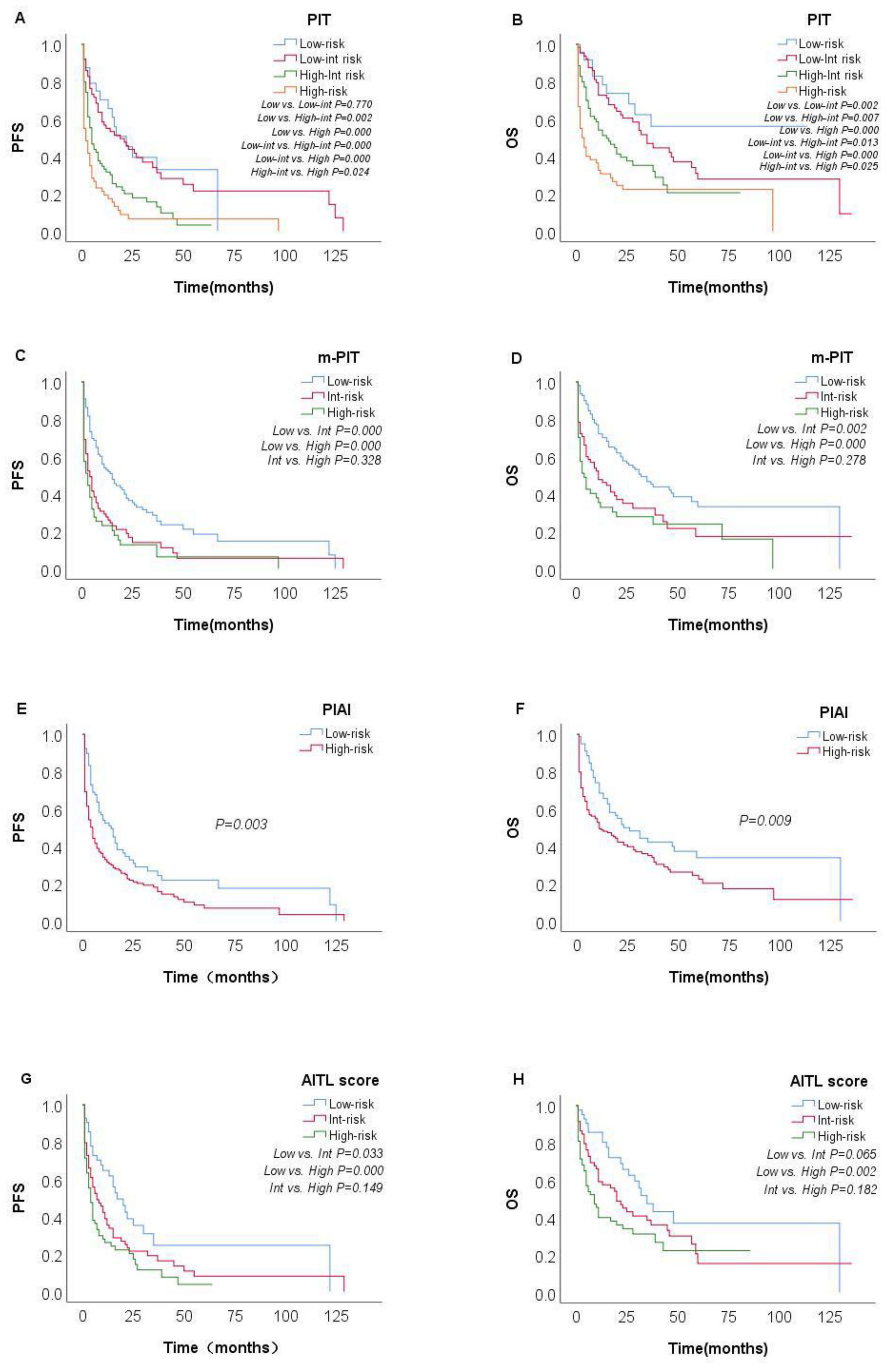
Survival probabilities of the overall AITL cohort. Kaplan–Meier curves show PFS and OS in patients with AITL stratified by PIT [**(A, B)** n=218], m-PIT [**(C, D)** n=222], PIAI [**(E, F)** n=231], and AITL score [**(G, H)** n=176].

Among the patient groups stratified by PIT (**Figures 4A, B**), there was no significant difference observed in OS and PFS between the low-risk and low-int risk group (P=0.226 and P=0.770). The m-PIT effectively discriminates only between low-risk groups (**Figures 4C, D**), with no significant difference found between int-risk and high-risk groups (P=0.955). Patients were stratified into only low- and high-risk groups based on PIAI (**Figures 4E, F**), with a significant difference observed between the two groups. The 2-year OS showed little numerical difference between the groups (low risk: 50.0% vs. high­risk: 40.7%), as did the 2-year PFS rates (low-risk: 32.7% vs. high-risk: 21.8%). The AITL score was predictive of PFS, although distinguishing the int-risk group from the other two cohorts in terms of OS according to the AITL score was challenging (**Figure 4H**). Even for PFS (**Figure 4G**), there was no significant difference between the int-risk and high risk group. These findings underscore the urgent need for a more finely stratified prognostic model capable of predicting both OS and PFS.

**Figure 5:**
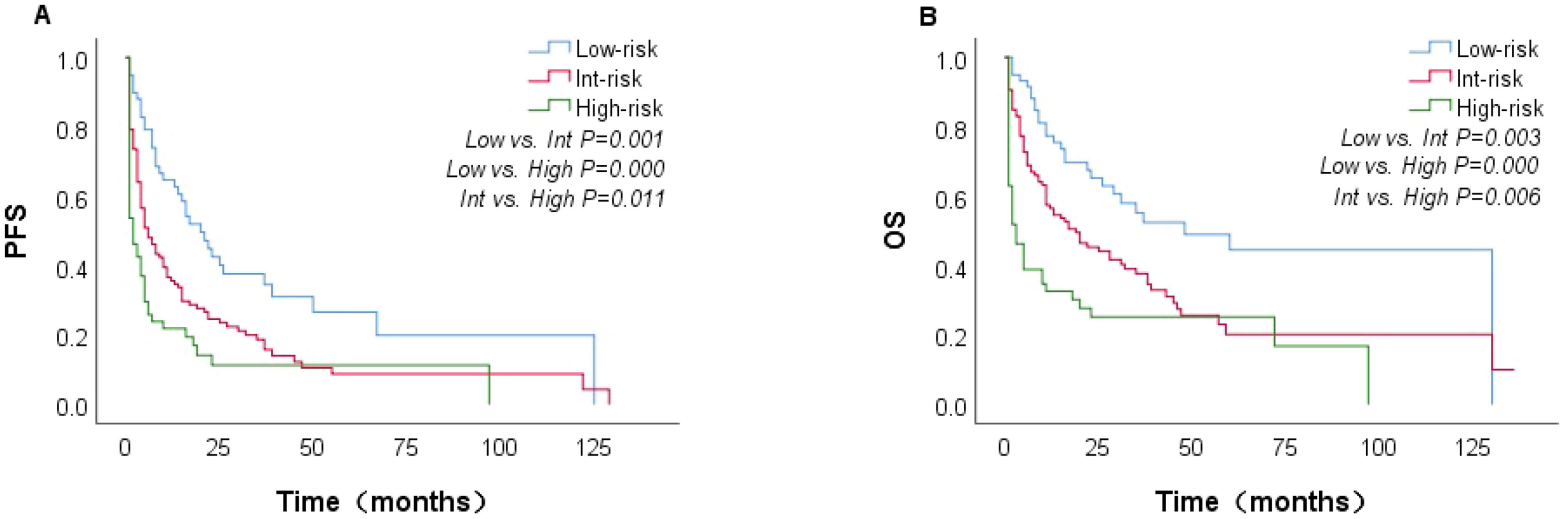
Kaplan–Meier curves showing PFS and OS in patients with AITL, stratified by the AITL model (n=221). PFS **(A)** and OS **(B)**.

In the univariate analysis, factors associated with inferior OS included age >60 years, ECOG performance status ≥ 2, anemia, NLR2 < 4.635, elevated β2M, elevated LDH, Alb < 3.5 g/dL, eosinophil < 0.02, DBIL > 6.8, hepatomegaly, serous cavity effusion, SUVmax > 12, *Ki-67* >45%, and CD20+ and EBV-DNA ≥ 500 copies/mL (**Table 1**). The multivariate analysis showed that only the following 4 factors retained independent prognostic value for OS: age >60 years, ECOG ≥2, elevated LDH and splenomegaly. Based on these 4 factors, we developed a novel prognostic score (AITL model) that stratified patients into low- (score between 0 and 1), intermediate-(score between 2 and 3), and high -risk (score 4). Patients classified as low-, intermediate-, and high-risk based on the AITL model had 2-year PFS estimates of 42.4%, 23.4%, and 0%, respectively (P<0.05, **Figure 5A**) and 2-year OS estimates of 63.6%, 42.1%, and 18.6%, respectively (P<0.05, **Figure 5B**). The new model (AITL model) therefore demonstrated predictive ability for both OS and PFS, with improved discriminatory power relative to previous models.

We additionally evaluated pathological features and imaging indices as possible prognostic factors, including CD20, CD30+, EBER - ISH, Ki-67 index, and SUVmax of the lymph node. In the univariate analysis, Ki-67 index > 45% (p = 0.005), CD20+ (P=0.026), and SUVmax of lymph node >12 (P=0.036) significantly influenced OS. However, in multivariate analysis, neither factor was found to be independent prognostic factor for OS. **Table 4** shows the adverse prognostic factors included in the AITL model and other models.

**Table 4:** Adverse prognostic factors in AITL.

Adverse prognostic factor	PIT	m-PIT	PIAI	AITL score	AITL model
Age ≥60 y	**+**	**+**	**+**	**+**	**+**
ECOG>2	**+**	**+**	**+**	**+**	**+** ^*^
LDH>ULN	**+**	**+**			**+**
Stage III-IV					
Extranodal sites≥2			**+**		
Bone marrow involvement	+				
B symptoms			**+**		
Splenomegaly					**+**
Thrombocytopenia (<150 000/mm3)			**+**		
Ki-67≥80%		**+**			
CRP>ULN				**+**	
β2M>ULN				**+**	

*: ECOG≥2 in AITL model and ECOG>2 in the other 4 models; CRP>ULN: C-reactive protein>5; β2M>ULN: β2M>3.

## 4 Discussion

AITL, the most common subtype of lymph node TFH lymphoma, is characterized by the specific expression of TFH markers such as CD10, BCL6, PD-1, CXCL13 and ICOS. Patients with AITL frequently harbor alterations in epigenetic genes such as *TET2*, *DNMT3A*, *IDH2*, and *RHOA* (20). The pathogenesis of AITL is complex, involving close interactions between tumor cells, the immune microenvironment, and EBV (21). It is hypothesized that dysregulation of the immune microenvironment and EBV infection contribute to immune escape and promote the survival of infected cells, thereby driving disease progression (11, 22). AITL occurs more frequently in older men, with a median age of 63 years. The majority of patients (88.3%) presented with advanced-stage disease (III/IV) at the time of diagnosis. Patients often present with systemic lymphadenomegaly due to immune dysregulation. Extranodal involvement is not uncommon in AITL, with 19.9% of patients in our cohort having extranodal involvement at more than two extranodal sites. Bone marrow involvement was present in 27.9% of patients, and 58.4% had splenic involvement.

Most patients (43.3%) were treated with CHOP-like regimens, and only 4.8% received CHOPE regimens, likely because of the older age of patients in our cohort. Consistent with prior reports (23), the addition of etoposide in our study did not improve the OS and PFS (P=0.545 and P=0.943, respectively), indicating that intensive regimens may not improve the prognosis of patients with AITL. In recent years, epigenetic-modifying therapies and gemcitabine-based chemotherapy have been explored in several studies for PTCL with the aim of improving clinical outcomes in untreated patients (17, 24, 25). Epigenetic drugs have demonstrated favorable short-term effects and longer duration of response in AITL (26, 27). Furthermore, the efficacy of chidamide in combination with PET regimens (CPET) has been evaluated in several clinical trials (18), further supporting the higher mutation frequency of *TET2* and *DNMT3A* in AITL. In our study, 71 patients with untreated AITL received epigenetic drugs in combination with chemotherapy; however, no significant difference in OS and PFS with or without epigenetic drugs (P=0.743 and P=0.704, respectively) was observed. We also found no significant benefit from PET-like regimens compared with CHOP-like regimens in AITL patients. A small proportion of patients (43 cases) were treated with gemcitabine-based chemotherapy. Consistent with previous studies (28, 29), the gemcitabine- based regimen was not superior to CHOP as front-line therapy in patients with untreated AITL in our study. Only 9 patients in our study underwent consolidative ASCT, and no patients were treated with brentuximab vedotin, which may partly reflect the older age and economic constraints of our cohort. Although new therapies, including chidamide, azacitidine, and gemcitabine, offer more treatment options for patients with AITL, we did not observe significant differences in outcomes. The efficacy of CHOP+X and ASCT after first remission for patients with AITL still needs further investigation.

The prognosis of patients with AITL is dismal. Our study analyzed the prognosis of a large retrospective AITL cohort, with 5­year OS and PFS estimates of 26.6% and 11.9%, respectively. The outcomes were slightly different to those reported in a large population-based study from the Swedish Lymphoma Registry (5-year OS and PFS estimates of 32% and 20%, respectively) (30), confirming adverse clinical outcomes. Although the majority of patients with AITL were sensitive to chemotherapy, the response duration was typically brief, and frequent relapses led to a low survival rate. We also found that nearly two-thirds (151 cases) of patients experienced disease progression within 12 months. The identification of reliable prognostic indicators is crucial for selecting appropriate treatment regimens, and there is an urgent need for prognostic models that can specifically stratify AITL.

To date, few studies have explored prognostic factors in AITL. Our study validated the predictive value of the PIT, m-PIT, PIAI, and AITL score. PIT (31)is a prognostic model specific to PTCL-NOS. Went et al. (32) proposed m-PIT after considering pathological factors, and reported that m-PIT outperformed PIT in terms of prognostic prediction. The clinical characteristics and genetic background of AITL differ significantly from those of PTCL-NOS, and the prognostic model developed for PTCL-NOS is not applicable to AITL (33). The PIAI was also validated in our cohort (P < 0.05). However, it was only categorized into two groups, namely, low-risk and high-risk, with the 2-year OS (50.0% vs 40.7%) and PFS (32.7% vs 21.8%) between the two groups being numerically close. Moreover, this classification approach exhibited limitations in accurately stratifying high-risk patients based on their risk scores. Ranjana H (16) proposed the AITL score and identified β2M and CRP as independent prognostic factors for PFS in a limited cohort of 96 patients. This model was not well validated in our cohort, and the int-risk group was not significantly different from the other groups. There have been few follow-up studies for PIAI and AITL score, and these have not yet been widely applied in clinical practice. A recent Asian multicenter study (34) found that age > 60 years, bone marrow involvement, total WBC >12 × 10^9^/L, and elevated LDH were associated with inferior outcome in AITL; on this basis, they proposed a new model (AITL-PI). Additionally, the AITLI model (35) was developed based on age >60 years, albumin <30 g/L, Ki-67 rate ≥70%, and a positive Coombs test. Some studies (12, 13, 36) have focused on the importance of metabolic parameters in PET/CT, finding that baseline TLG (total lesion glycolysis), SUVmax of spleen, and SUVmax of extranodal lesions are strong predictors of AITL. However, the number of cases included in these studies was relatively small, and the application of these indicators remains controversial. This further highlights the lack of effective prognostic models for patients with AITL.

We identified age >60 years, ECOG≥2, elevated LDH as independent prognostic factors associated with OS; these have also been recognized as strong influencing factor in previous studies (20, 35). The novel model (AITL model) combining four variables (age >60 years, ECOG≥2, elevated LDH and splenomegaly) stratified patients into low-, intermediate-, and high-risk groups. The AITL model demonstrated strong power of classification based on risk score and good predictive value for both PFS and OS. In a large data cohort of 221 patients, we identified novel prognostic factors and developed a new prognostic model. The 2-year OS was 63.6%, 42.1%, and 18.6% for the low-, intermediate-, and high-risk groups, respectively. The 2-year PFS was 42.4%, 23.4%, and 0%, respectively. Patients classified as high-risk according to the AITL model all experienced disease progression within 2 years. In comparison, the 2-year PFS was 6.6% to 21.8% for patients with high-risk PIT, m-PIT, PIAI, and AITL scores. This indicates that the AITL model has a superior ability to identify patients who are insensitive to chemotherapy or have a short duration of response, showing good discriminatory ability. Lymphoma involvement of the spleen is common, and the lymph nodes and spleen are the most common primary sites of PTCL (37), as predominantly characterized by splenomegaly on imaging. This study comprised a higher percentage of patients with splenomegaly—58.4%, compared with 46.8% in previous studies (2, 18). This discrepancy may be attributable to our inclusion of splenomegaly based on imaging reports (US, CT, PET/CT). PET/CT shows sensitivity in detecting splenic involvement in patients with lymphoma, and some studies have suggested that SUVmax of the spleen can be used as a reference index for determining the prognosis of patients with AITL (14, 36). In conclusion, patients with splenomegaly tended to demonstrate worse OS than those without splenomegaly. The four factors identified in our study are common and practical for use in clinical applications. Our findings suggest that the model can effectively identify high-risk patients with AITL and accurately predict survival outcomes.

Few studies have attempted to identify pathological prognostic factors in AITL. This study sought to evaluate the potential prognostic value of CD20, CD30, and the Ki-67 index. We found that CD20 positivity and Ki-67 >45% were associated with poor OS; however, these factors did not show independent prognostic value for OS in the multivariate analysis. We analyzed PET/CT parameters and found that high lymph node SUVmax (SUVmax >12) was indicative of shorter OS, although not statistically significant (P=0.183). Furthermore, a high EBV viral load at diagnosis was associated with shorter OS and emerged as a key marker of poor prognosis, while positive EBER-ISH did not significantly affect survival, consistent with previous studies (38, 39).

The main limitation of this study is that it is a retrospective analysis. Our cohort spanned 10 years, from 2014 to 2023 (all post-2010, i.e., after FDA approval of romidepsin for disease relapse, reflecting an era of increased use of novel medications). Furthermore, owing to the limitations of the retrospective study itself, the new model was only internally validated. The proposed model still requires further evaluation and validation in prospective or multi-center cohorts. Although the first-line therapy varied among the cohort, there was no significant difference in survival at different periods (2014–2018 vs. 2019–2023).

In conclusion, the long-term outcomes for patients with AITL treated with contemporary chemotherapeutic regimens remain unsatisfactory. In terms of therapeutic outcomes, there was no significant difference between anthracycline- or epigenetic- or gemcitabine-based regimens, highlighting the need for further exploration of standardized therapeutic regimens. Splenomegaly at initial diagnosis was identified as an independent poor prognostic factor for OS. The new model (AITL model) demonstrated strong classification and predictive power for both OS and PFS in patients with AITL.

## Data Availability

The original contributions presented in the study are included in the article/supplementary material. Further inquiries can be directed to the corresponding author.
